# Defining ‘actionable’ high- costhealth care use: results using the Canadian Institute for Health Information population grouping methodology

**DOI:** 10.1186/s12939-019-1074-3

**Published:** 2019-11-10

**Authors:** Maureen Anderson, Crawford W. Revie, Henrik Stryhn, Cordell Neudorf, Yvonne Rosehart, Wenbin Li, Meriç Osman, David L. Buckeridge, Laura C. Rosella, Walter P. Wodchis

**Affiliations:** 1 Department of Health Management, University of Prince Edward Island, Charlottetown, Prince Edward Island Canada; 2 Department of Community Health and Epidemiology, College of Medicine, University of Saskatchewan, Saskatoon, Saskatchewan Canada; 3 Department of Computing and Information Sciences, University of Strathclyde, Glasgow, Scotland; 4 Population and Public Health, Saskatchewan Health Authority, Saskatoon, Saskatchewan Canada; 5 Canadian Institute for Health Information, Ottawa, Ontario Canada; 6 Saskatchewan Health Quality Council, Saskatoon, Saskatchewan Canada; 7 Institute for Clinical Evaluative Sciences, Toronto, Ontario Canada; 8 Department of Epidemiology, Biostatistics, and Occupational Health, McGill University, Montreal, Quebec Canada; 9 Public Health Ontario, Toronto, Ontario Canada; 10 Dalla Lana School of Public Health, University of Toronto, Toronto, Ontario Canada; 11 Institute of Health Policy, Management and Evaluation, University of Toronto, Toronto, Canada

## Abstract

**Background:**

A small proportion of the population consumes the majority of health care resources. High-cost health care users are a heterogeneous group. We aim to segment a provincial population into relevant homogenous sub-groups to provide actionable information on risk factors associated with high-cost health care use within sub-populations.

**Methods:**

The Canadian Institute for Health Information (CIHI) Population Grouping methodology was used to define mutually exclusive and clinically relevant health profile sub-groups. High-cost users (> = 90th percentile of health care spending) were defined within each sub-group. Univariate analyses explored demographic, socio-economic status, health status and health care utilization variables associated with high-cost use. Multivariable logistic regression models were constructed for the costliest health profile groups.

**Results:**

From 2015 to 2017, 1,175,147 individuals were identified for study. High-cost users consumed 41% of total health care resources. Average annual health care spending for individuals not high-cost were $642; high-cost users were $16,316. The costliest health profile groups were ‘long-term care’, ‘palliative’, ‘major acute’, ‘major chronic’, ‘major cancer’, ‘major newborn’, ‘major mental health’ and ‘moderate chronic’. Both ‘major acute’ and ‘major cancer’ health profile groups were largely explained by measures of health care utilization and multi-morbidity. In the remaining costliest health profile groups modelled, ‘major chronic’, ‘moderate chronic’, ‘major newborn’ and ‘other mental health’, a measure of socio-economic status, low neighbourhood income, was statistically significantly associated with high-cost use.

**Interpretation:**

Model results point to specific, actionable information within clinically meaningful subgroups to reduce high-cost health care use. Health equity, specifically low socio-economic status, was statistically significantly associated with high-cost use in the majority of health profile sub-groups. Population segmentation methods, and more specifically, the CIHI Population Grouping Methodology, provide specificity to high-cost health care use; informing interventions aimed at reducing health care costs and improving population health.

## Introduction

Increasing health care costs are challenging health care systems in Canada and around the world. Health care expenditure in nearly every developed country meets or exceeds 10% of gross domestic product [31]. Evidence has long demonstrated that a small proportion of the population (< 10%) accounts for the majority (50–70%) of total health care spending [7, 20, 21, 26]; individuals commonly referred to as ‘high-cost users’.

High-cost users are a heterogeneous population. From 1985 (*n* = 79) to 2018 (*n* = 1198) the number of PubMed citations involving the term ‘high-cost use’ has increased over 15 times [17]. In general, previous studies have found high-cost health care use to be associated with: 1) complex, multiple chronic conditions (multi-morbidity), 2) catastrophic illness (for example, motor vehicle accident or major cancer), 3) high costs at end-of-life, 4) mental health and addictions, 5) institutional living, and, 6) various indicators of lower socio-economic status (for example, food insecurity and poverty) – including combinations thereof. A recent systematic review on high-cost health care users identified similar patterns: multi-morbidity, mental health and addictions, increasing age, end-of-life care and socio-economic status were the predominant factors associated with high-cost use across 55 countries globally [29].

One of the pioneers in understanding high-cost health care use is Dr. Jeffrey Brenner and his team in Camden, New Jersey. They found high-cost users were predominately located in two high-rise urban apartment buildings in an impoverished neighbourhood in Camden City. Case management strategies, such as providing ‘wrap-around’ care for complex patients, aimed specifically at the identified population both improved health outcomes and reduced cost [6, 12].

When public health action is focused on a relatively small population, such as the one in Camden, New Jersey [6, 12] it is easier for the insurers to describe and create policies aimed at improving health outcomes and reducing inappropriate health care costs. For example, an interdisciplinary team of care providers in Camden were able to co-ordinate their services within a specific geographical location and a relatively homogenous population (low socio-economic status). However, when a population is more diffuse and broad, as is the case with publicly insured provincial populations in Canada, describing and creating policies aimed at reducing high-cost health care use are more difficult.

High-cost user studies can overlook health equity considerations, even when segmenting the population into meaningful sub-groups based on disease profiles [11, 16]. To that end, we aimed to understand, by specific mutually exclusive health profile groups, factors driving high-cost health care use within each health profile group under study. By defining ‘high-cost use’ within sub-groups, we achieve better specificity to the high-cost definition. For example, if one were to define ‘high-cost use’ as those in the > = 90th percentile of spending in the provincial population overall, factors that are related to high-cost use in general would be understood; however, nuances of factors that may be associated with high-cost use within a specific subset, such as acute disease versus chronic disease, would be lost.

### Study objectives

1) To describe and define high-cost health care use in the provincial population of Saskatchewan, Canada and, 2) From the perspective of a provincial government funding health care in Canada, to explore risk factors associated with high-cost health care use within specific health profile groups.

In the current study, ‘actionable’ is defined as quantitatively understanding risk factors associated with high-cost use, within sub-populations, including variables that are typically included in modelling high-cost use but less amenable to change (for example, age) with variables that may be more amenable to change and therefore ‘actionable’ through targeted programmatic or policy interventions (for example, socio-economic status).

Achieving equity in health is one of the key drivers of this research; high-cost health care users are more costly to the health care system, but, why is not necessarily clear. Previous research has demonstrated that disease burden is higher amongst the poor and poverty leads to poor health outcomes. Health care systems need to consider and address the underlying social determinants of health of the populations they serve. It is compelling that health management organizations in the United States, where cost and profits are considerations, have started investing in housing as a strategy to reduce health care expenditures [13, 18, 30].

In order to achieve study objectives, we used a population segmentation method recently developed by the Canadian Institute for Health Information (CIHI). The population grouping methodology (‘Pop Grouper’) builds clinical and demographic profiles for each person in a population, including health system non-users.

CIHI’s population grouping methodology enables health system planners and policy-makers to use evidence to support decision-making. This assists CIHI and its clients monitor population health and diseases, predict health care utilization patterns and explain variations in health care resource use, provide a foundation for funding models, and, facilitate standardization of populations for inter-provincial analyses [10] (see Methods and Additional file 1).

Given rising health care costs, increased demand from growing elderly populations with multi-morbidity, and, health consuming more and more of the total gross domestic product in many countries around the world, policy makers and health researchers have, reasonably, been looking to the population of ‘high-cost users’ for cost savings.

#### Theoretical framework

As this research uses health care utilization data, it is important to understand the theoretical framework surrounding health care utilization in general. The ‘Behavioural Model of Health Services Use’ developed by RM Andersen in 1968 (updated in 1995 and renamed the ‘Andersen Health care Utilization Model’) is considered foundational work in this area [3].

This conceptual model describes the factors that lead to the use of health services. According to the model, there are different dynamics that affect an individual’s health care utilization. Andersen categorizes predictors into three categories: need, enabling and predisposing factors [3, 5]. Briefly, *predisposing* characteristics are those that predispose individuals to use or not use health care services (such as age, sex, ethnicity). *Enabling* characteristics are those that either increase or decrease the likelihood of health care service use (such as trust of health care system, income level). *Need* characteristics are those related to health care service, both perceived and actual need, such as the presence of chronic conditions.

The model makes a distinction between equitable and non-equitable access to health care services. Equitable access relates to predisposing factors and need. Inequitable access relates to predisposing and enabling factors. For example, an individual who believes Western medical health care services are beneficial to their perceived need are more likely to seek care; however, the ability to access services might vary based on ethnicity, sexual orientation, economic status, and other factors.

To the best of our knowledge, this is the first study to segment the population into clinically meaningful sub-groups, define high-cost use within each sub-group and include a measure of socio-economic status in multivariable regression models.

## Methods

Due to the availability of administrative health data through in-kind support from host institutions and data-sharing agreements, this study focuses on the provincial population of Saskatchewan, Canada (population ~ 1.2 million), a Canadian province with a central provincial health insurer. All Saskatchewan residents receive provincial health care benefits - with the exception of less than 1% of the population for whom benefits are provided by the federal government (members of the armed forces and federal penitentiary inmates) [8]. Every provincial resident with a valid health service number (‘health card’) for at least 1 day from April 1, 2015 to March 31, 2017 was eligible for study.

### Population segmentation method

The Canadian Institute for Health Information (CIHI) population grouping methodology was used to segment the study population into clinically meaningful and mutually exclusive health profile groups [10] (Additional file 1). Throughout the world, diagnosis-related grouping (DRG) methodologies are used to group alike disease conditions. American DRGs are readily available for use in Canada; however, these models have two significant drawbacks: 1) not designed using Canadian data and 2) they are intellectual property of the company that produce them, and, as such, are relatively expensive to purchase.

In brief, CIHI’s population grouping methodology uses a combination of administrative health databases and provincial health registry systems to ‘tag’ each resident with any of the 239 health conditions. These binary tags (0/1) form the ‘building block’ of the grouping methodology and are not mutually exclusive; an individual can have any number of applicable health conditions. The presence of health conditions are determined by linking data from hospitalizations, physician visits, hospital day surgeries, emergency department visits and long-term care. The method uses 24 months of data to determine health conditions (*n* = 239), branches (*n* = 164) and health profile groups (*n* = 16). Please see ‘Additional file 1’ for details.

Unlike common comorbidity measures such as the Charlson or Elixhauser indexes (Southern, 2004) the Pop Grouper does not require an individual to have been hospitalized; Case Mix Groups (a DRG) similarly require the individual to have been hospitalized. Pop Grouper categories are available for every resident, including non-users of the health care system. Unlike proprietary DRGs or other population segmentation methods, Pop Grouper was developed in Canada, underwent extensive validation exercises using Canadian data and its development made use of Canadian clinical content experts [10].

During the course of the study, we identified a further ‘health profile group’, ‘Long-term care (LTC) resident’; the RAI-LTC 2.0 (see ‘Databases’ for details) was used to identify long-term care residents. After health profile grouping, logistic models were constructed for each profile group to understand factors associated with ‘high-cost health care use’ (defined as > = 90th percentile within health profile groups). Independent variables in the models were those commonly available in administrative health databases (demographic and health care utilization data). We used an area-based measure of health inequity in our modelling. In Canada, a measure developed by the Institute National Santé Publique du Quebec divides the population, at the neighbourhood level, into deprivation quintiles. The five categories segment the population into the most privileged (quintile 1) to the most deprived (quintile 5). Total deprivation is a combination of total household income, education and employment levels [15].

We made use of Andersen’s behavioural model and theoretical framework for health services use in describing predictors of interest [3, 5]. Where the data is in routine administrative health data, these variables are in the models.

### Databases

Detailed descriptions of Saskatchewan Health databases are available elsewhere [8]. In brief, demographic characteristics, location of residence, and neighbourhood income quintile were extracted from the *Personal Health Registration System (PHRS)* for individuals with > = 1 day of valid health insurance coverage within the study period. Hospital data extracted from the *CIHI-Discharge Abstract Database (DAD)* includes inpatient and day surgery records for the province of Saskatchewan. Out-of-province hospitalizations for Saskatchewan residents were included; transfers were included but ‘counted’ as one hospitalization. The International Classification of Diseases (ICD), 10th revision, Canadian Version (ICD-10-CA) was used in the DAD to record up to 25 diagnoses at discharge, including the primary responsible diagnosis for that hospitalization. Data on physician services are contained in the *Medical Services Claims Database*. Physicians paid on a fee-for-service basis submit billing claims to the provincial health ministry; a single diagnosis using a three-digit ICD-9 code is on each claim. The same single diagnosis is on every claim submitted for a single visit – multiple service claims by the same physician, same patient, and same day counted as one ‘visit’. Salaried physicians can submit billing claims for administrative purposes (shadow billing); however, claims from salaried physicians involve under-reporting resulting in fewer physician claims in the data than in practice [24]. The provincial *Resident Assessment Instrument-Minimum Dataset* for long-term care facilities (RAI-LTC 2.0) defined residents of long-term care facilities. Home care data is available in the *Resident Assessment Instrument-Minimum Dataset* for home care facilities (RAI-HC). Emergency department (ED) visit data was recorded in *National Ambulatory Care Reporting System* (NACRS); however, for the study period < 50% of the provincial emergency departments were NACRS-reporting facilities resulting in under-reporting of ED visits. Prescription drug data was used to calculate total government paid prescription drug costs at the individual-level (see ‘Outcome’), but, drug data was not extracted as health care utilization.

Death was defined in a ‘derived death file’ (combination of death data from various administrative health databases) created by the Saskatchewan Health Quality Council for research purposes. Nominal variables were removed (i.e.) name and health services numbers and, data sets linked at the individual-level using a unique non-identifiable number generated by eHealth Saskatchewan.

### Outcome

We calculated total health care costs for each individual for the study period. Health care costs were a sum, at the individual-level, of total costs associated with hospitalizations (both in-patient and day surgery), physician visits, emergency department visits, long-term care beds and prescription drugs. We accounted for inflation by adjusting all health care costs to the year 2015. The CIHI Resource Intensity Weight (RIW) value at the patient-level was multiplied by the CIHI derived value ‘cost of a standard hospital stay’ to calculate hospitalization costs. In the absence of RIWs for LTC in Saskatchewan, we used a per-diem approach to calculate LTC costs (per-diem value calculated using LTC actual expenditure, resident fees and number of LTC beds). Calculations used total government-paid costs for each database. We assigned costing variables to their fiscal year of occurrence. Total health care costs attributed to each individual in the study population were calculated using the person-level costing methodology developed by Wodchis et al. Briefly, this methodology provides guidance on how to identify unit costs associated with individual health care utilization of emergency departments, hospitalizations, physician visits, long-term care and prescription drugs. The method includes the ability to combine these costs with utilization data from administrative health databases; providing a measure of direct health care costs incurred by government. Each of the administrative health databases calculates cost in a different way – we then unified the calculation across the databases by combining them as the ‘total cost per person’.

In the current study, we defined high-cost status within each health profile group. Study cohort members considered ‘high-cost’ vary by health profile group; however, we consistently defined high-cost as the 90th percentile of total cost within the health profile group. Total insurance coverage length of time was not available for analysis limiting our ability to account for death/relocation during the study period (individuals with less follow-up time would have lower costs, see Limitations). For each health profile group (*n* = 16) ‘high-cost users’ were uniquely identified within the health profile group as those individuals exceeding the 90th percentile of group-specific total health care costs (*n* = 10% within each health profile group). The health profile groups with the highest total costs (*n* = six, excluding palliative care and long-term care residents) were modelled to assess factors associated with high-cost use: ‘Major acute’, ‘Major chronic’, ‘Major cancer’, ‘Moderate chronic’, ‘Major Newborn’ and ‘Other Mental Health’.

### Predictors

Health care utilization variables are, in most instances, directly related to health care costs. Comorbid conditions, age, sex, geographical location and socio-economic status variables are, in most instances, potentially confounding variables in the relationship between utilization and cost [29].

Demographic and socio-economic variables (geographic location, age, sex; *predisposing factors*; neighbourhood income quintile; *predisposing and enabling factors*) were defined as of study index date (April 1, 2015) and extracted from the provincial *Personal Health Registry System*. Chronic comorbid conditions were identified using the health condition ‘tags’ embedded within the Pop Grouper (*need factors*). Health care utilization variables - number of physician visits, number of emergency department visits, number of hospitalizations, home care visits, long-term care residency, length of stay in hospital and alternate level of care hospital days - were extracted from the relevant administrative health database, by fiscal year, for the duration of the entire study period (April 1, 2015 to March 31, 2017). ‘Alternate level of care’ hospital days refer to those days spent in hospital where the level of acuity is not needed for the patient, however, there does not exist a suitable place for the patient to be transferred to; most typically, individuals are awaiting a LTC bed [14].

We defined health profile groups using the CIHI Population Grouping Methodology software. A detailed description of this methodology precedes this section. We used relevant binary health condition ‘tags’ to define cancer, dialysis, mental health and neonatal intensive care conditions. Individuals ‘tagged’ with > = one of these health conditions were considered to have the relevant condition(s). We defined multiple chronic conditions using the same binary health conditions tags for minor, moderate and major chronic health profile groups. Based on descriptive analyses of the data, any individual with > = three of these chronic condition ‘tags’ were considered to have multiple chronic conditions. Categorical variables of health care utilization (‘high’ number of visits versus not) were defined as any individual > = 75th percentile of total study population health care utilization (this equated to: physician visits> = 23; emergency department visits > = one and hospitalizations > = one over the study period). The 75th percentile cut-off was used as descriptive statistics indicated the continuous count variables sharply rose at this value; thus marking a potential difference between ‘high’ and ‘low’ (subsequently modelled to understand the effect). See Table 1 for descriptive statistics of all study variables and Table 2 for Pop Grouper data  summary.

**Table 1:** Descriptive epidemiology, demographics and health care utilization variables, by cost category, study population, April 1, 2015 – March 31, 2017 (*n* = 1,175,147)

	Lowest 90%, Saskatchewan (*n* = 1,057,635)	Top 10%, Saskatchewan (*n* = 117,512)
Age		
18–79 years	1,022,482 (96.7%)	100,667 (85.7%)
80+ years	35,153 (3.3%)	16,845 (14.3%)
Sex		
Male	542,573 (51.3%)	49,906 (42.5%)
Female	515,062 (48.7%)	67,606 (57.5%)
Geographic location		
Urban	711,058 (67.2%)	77,693 (66.1%)
Rural	317,614 (30.0%)	36,656 (31.2%)
Missing	28,963 (2.7%)	3163 (2.7%)
Neighbourhood income		
1 (least affluent)	225,650 (21.3%)	26,426 (22.5%)
2	192,838 (18.2%)	21,918 (18.7%)
3	177,797 (16.8%)	19,685 (16.8%)
4	202,522 (19.2%)	22,363 (19.0%)
5 (most affluent)	176,755 (16.7%)	18,710 (15.9%)
Missing	82,073 (7.8%)	8410 (7.2%)
Health profile category		
Palliative	1456 (0.1%)	4754 (4.1%)
Major acute	19,010 (1.8%)	15,723 (13.4%)
Major chronic	20,292 (2.0%)	17,511 (14.9%)
Major newborn	1519 (0.1%)	1159 (1.0%)
Major mental health	11,031 (1.0%)	6581 (5.6%)
Major cancer	4803 (0.5%)	3908 (3.3%)
Moderate acute	63,528 (6.0%)	13,492 (11.5%)
Moderate chronic	87,826 (8.3%)	24,950 (21.2%)
Other cancer	4595 (0.4%)	1321 (1.1%)
Other mental health	58,587 (5.5%)	3794 (3.2%)
Obstetrics	22,399 (2.1%)	12,794 (10.9%)
Minor acute	424,832 (40.2%)	7087 (6.0%)
Minor chronic	146,122 (13.8%)	3731 (3.2%)
Healthy newborn	9601 (0.9%)	412 (0.4%)
Health system user, no health conditions	52,527 (5.0%)	83 (0.1%)
Health system non-user	129,507 (12.2%)	212 (0.2%)
Multi-morbidity (> = 3 conditions)		
Yes	96,570 (9.1%)	54,392 (46.3%)
No	961,065 (90.9%)	63,120 (53.7%)
Died during study period		
Yes	9244 (0.9%)	11,686 (9.9%)
No	1,048,391 (99.1%)	105,826 (90.1%)
Home care client		
Yes	47,228 (4.5%)	34,357 (29.2%)
No	1,010,407 (95.5%)	83,155 (70.8%)
Number of hospitalizations		
(mean/SD)	0.22 (0.6)	2.1 (2.1)
Length of stay (LOS) in hospital (days)		
(mean/SD)	0.72 (7.1)	13.0 (33.1)
Alternate level of care		
Yes	2521 (0.2%)	4340 (3.7%)
No	1,055,114 (99.8%)	113,172 (96.3%)
Mental health condition		
Yes	108,044 (10.2%)	29,795 (25.4%)
No	949,591 (89.8%)	87,717 (74.7%)
Dialysis		
Yes	1657 (0.2%)	3287 (2.8%)
No	1,055,978 (99.8%)	114,225 (97.2%)
Emergency department visits		
mean/SD	0.2 (0.6)	0.8 (2.4)
Family physician visits		
mean/SD	4.4 (5.6)	12.8 (12.4)
Specialist physician visits		
mean/SD	2.7 (6.1)	16.0 (23.2)
Total physician visits > = 23/yr		
Yes	206,751 (19.6%)	96,693 (82.3%)
No	850,884 (80.5%)	20,819 (17.7%)
History of hospitalizations > = 1/yr		
Yes	41,575 (3.9%)	59,502 (50.6%)
No	1,016,060 (96.1%)	58,010 (49.4%)
Average annual health care cost ($)		
(mean/SD)	$642 ($895)	$16,316 ($23,992)
Total health care cost ($, %)	$2,049,772,060 (58.7%)	$1442,545,027 (41.3%)

**Table 2:** Mean health care costs, by health profile category and high-cost use, Saskatchewan, excluding long-term care residents, April 1, 2015 to March 31, 2017 (*n* = 1,175,147)

Health profile category^a^	n	Cost per person (mean, SD)
Palliative	6210	$30,301 (39,692)
Major Newborn	2678	$14,714 (39,401)
Major Acute	34,733	$12,062 (26,737)
Major Chronic	37,803	$11,663 (23,434)
Major Cancer	8711	$9652 (15,886)
Major Mental Health	17,612	$8389 (17,655)
Moderate Chronic	112,776	$3788 (7064)
Other Cancer	5916	$3782 (6320)
Obstetrics	35,193	$3489 (4273)
Moderate Acute	77,020	$2843 (4820)
Healthy Newborn	10,013	$2023 (1669)
Other Mental Health	62,381	$1442 (3306)
Minor Chronic	149,853	$918 (2188)
Minor Acute	431,919	$518 (1731)
Health System User, no health conditions	52,610	$160 (1221)
Health System non-user	129,719	- (−)

^a^Mutually exclusive health profile categories assigned by highest resource intensity April 1, 2015 to March 31, 2017

### Statistical analyses

Following univariate and bivariate analyses, we used multivariable regression modelling to delineate factors associated with high-cost use; as defined within each health profile group. Logistic regression model effect selection was achieved by limiting the number of covariates to those contributing most to outcome measures by choosing the model with the smallest Akaike’s information criterion (AIC) [1]. We explored all interaction terms analytically; only those deemed biologically plausible by clinician contributors and previous studies were included in the models. Where missing values were present in the PHRS (income quintile and location of residence), a categorical value of ‘missing’ was created in order to allow for sensitivity analyses, with and without the subjects with missing data. Sensitivity analysis, with and without missing data, were conducted. All analyses were conducted using SAS© Enterprise Guide version 7.1 [23].

The study proposal underwent ethical review and approval by the University of Saskatchewan Biomedical Research Ethics Board and the University of Prince Edward Island Research Ethics Board for research involving human subjects.

## Results

**Fig. 1:**
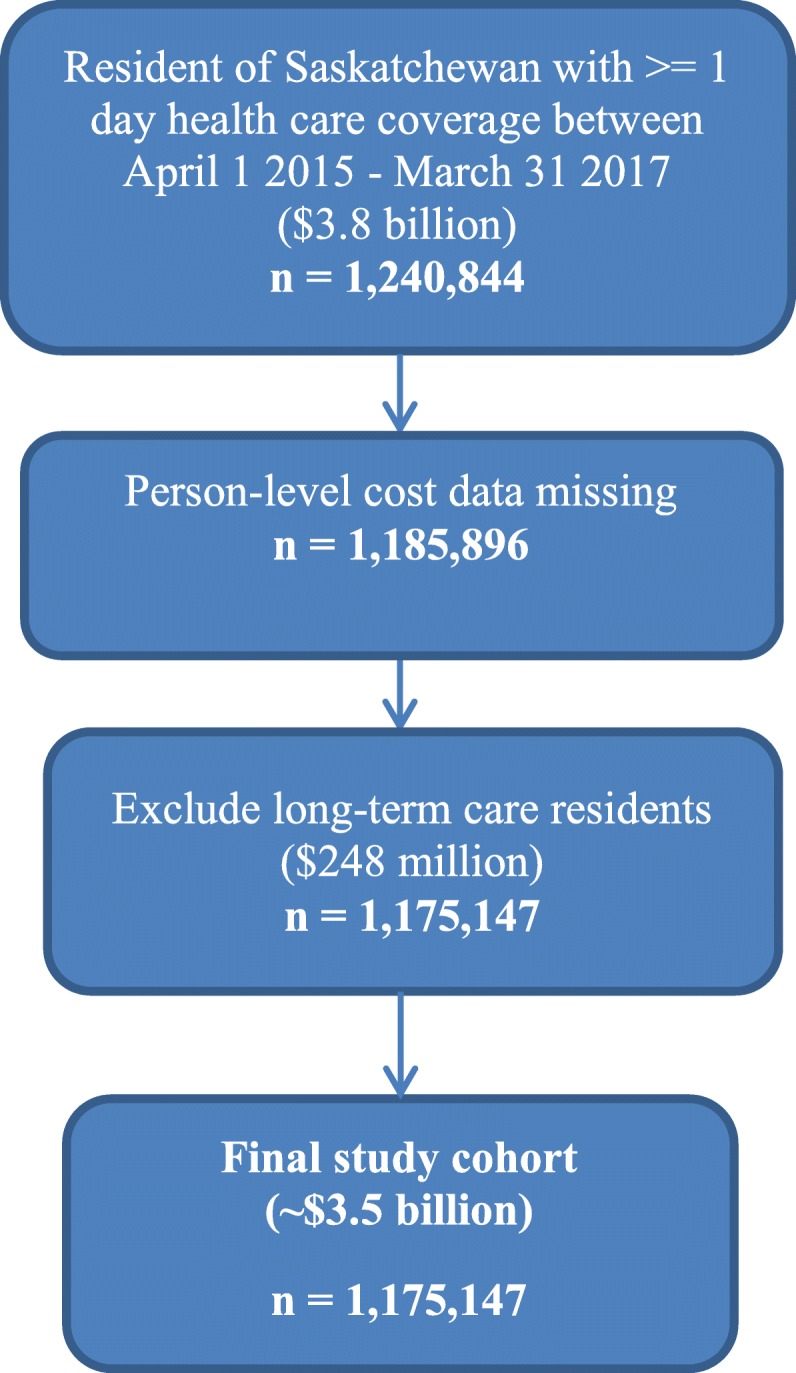
Study cohort inclusion/exclusion criteria

We identified a total of 1,175,147 individuals, residents of Saskatchewan, excluding residents of long-term care, with health insurance coverage of at least 1 day from April 1, 2015 to March 31, 2017 and person-level costing data (Fig. 1). Regardless of health condition, LTC residents were consistently high-cost health care users and therefore excluded from further study. In descriptive analyses of the provincial population, high-cost health care users (*n* = 117,512) were more likely to be older (80+ years), female, residents of rural Saskatchewan, lower income, have more than one chronic condition and die during the study period compared to non-high cost users. Compared to non-high cost users, high-cost users were more likely to have higher health care utilization, such as, home care services, be hospitalized (with longer lengths of stay and more ‘alternate level of care’ hospitalizations), visit the emergency department and have increased physician visits (Table 1); these findings varied by each health profile group (data not shown).

In terms of health profile groups, there were differences between high-cost users within groups compared to general population high-cost users. The groups ‘minor acute disease’, ‘health system non-user’, ‘minor chronic disease’ and ‘moderate chronic disease’ accounted for the majority of the study population. The groups ‘moderate chronic’, ‘major chronic disease’, ‘major acute disease’, ‘moderate acute’, and ‘obstetrics’ accounted for the majority (71.9%) of high-cost users. Overall, high-cost users in Saskatchewan comprised 10% of the study population but accounted for 41% of total health care costs in the study period. The following summarizes data by health profile group.

### Major chronic disease health profile group

Risk factors associated with high-cost use were assessed for individuals categorized as ‘major chronic disease’ over the study period. Using available variables and taking known risk factors for high-cost use into account, low income was significantly associated with high-cost health care use (OR = 29.4; 95% CI: 19.4–44.7); low income interacted with length of hospital stay exceeding 3 days in the study period. Individuals who did not have low income but did have a length of stay > = 3 days had a lower risk of high-cost health care use compared to individuals with low neighbourhood income and a length of hospital stay > = 3 days (OR: 15.3; 95% CI: 12.9–18.1) (Table 3).

**Table 3:** Logistic regression models comparing high-cost health care users and not high-cost use, excluding long-term care residents, by health profile category, Saskatchewan, April 1, 2015 to March 31, 2017 (*n* = 1,175,147). High-cost use within major chronic health profile group, odds ratios (Total *n* = 37,803; High-cost users *n* = 3781)

Focus setting	Comparison setting	OR	95% CI
Home care (=yes)	Home care (=no)	1.96	1.82–2.12
LOS = y; low income = y	LOS = n; low income = y	29.42	19.37–44.69
LOS = y; low income = n	LOS = n; low income = n	15.25	12.86–18.09
Multi chronic dx = y; High Dr. visits = y	Multi chronic dx = y; High Dr. visits = n	4.99	3.46–7.23
Multi chronic dx = n; High Dr. visits = y	Multi chronic dx = n; High Dr. visits = n	2.25	1.54–3.29

### Moderate chronic disease health profile group

Under the ‘moderate chronic disease’ health profile group, those with low neighbourhood income and high numbers of hospitalizations were more likely to be high-cost users, compared to low numbers of hospitalizations and not low income (OR = 4.8; 95% CI: 4.3–5.2) (Table 4).

**Table 4:** Logistic regression models comparing high-cost health care users and not high-cost use, excluding long-term care residents, by health profile category, Saskatchewan, April 1, 2015 to March 31, 2017 (*n* = 1,175,147). High-cost users within moderate chronic health profile group, odds ratios (Total *n* = 112,776; High-cost users *n* = 11,277)

Focus setting	Comparison setting	OR	95% CI
Home care (=yes)	Home care (=no)	2.04	1.94–2.14
Mental health (=yes)	Mental health (=no)	1.35	1.28–1.43
Hx of hosp = y; low income = y	Hx of hosp = n; low income = y	4.76	4.32–5.23
Hx of hosp = y; low income = n	Hx of hosp = n; low income = n	4.06	3.88–4.25

### Major acute disease health profile group

In the major acute disease health profile group, high-cost use was associated with long hospital stays (OR: 35.5; 95% CI: 26.8–47.1). In addition, among those with multiple chronic conditions and high numbers of physician visits had a reduced risk of high-cost health care use (OR: 2.1 versus OR: 3.8; 95% CI: 2.3–6.2) (Table 5).

**Table 5:** Logistic regression models comparing high-cost health care users and not high-cost use, excluding long-term care residents, by health profile category, Saskatchewan, April 1, 2015 to March 31, 2017 (*n* = 1,175,147). High-cost users within major acute disease health profile group (Total *n* = 34,733; High cost users *n* = 3473)

Focus setting	Comparison setting	OR	95% CI
Length of hospital stay > = 3 days	Length of hospital stay < 3 days	35.5	26.8–47.1
Multi chronic dx = y; High Dr. visits = y	Multi chronic dx = n; High Dr. visits = y	2.1	1.9–2.3
Multi chronic dx = y; High Dr. visits = n	Multi chronic dx = n; High Dr. visits = n	3.8	2.3–6.2

### Major cancer disease health profile group

In the major cancer health profile group, high-cost use was associated with health care utilization variables (emergency department, home care hospitalizations and length of stay) and the presence of multiple chronic conditions (Table 6).

**Table 6:** Logistic regression models comparing high-cost health care users and not high-cost use, excluding long-term care residents, by health profile category, Saskatchewan, April 1, 2015 to March 31, 2017 (*n* = 1,175,147). High-cost users within major cancer health profile group (Total *n* = 8711; High-cost users *n* = 872)

Predictor	Odds ratio	95% CI
Length of hospital stay > = 3 days (=yes)	23.11	13.82–38.65
High emergency department visits (=yes)	1.59	1.36–1.85
History of hospitalization (=yes)	1.61	1.26–2.05
Home care (=yes)	2.36	2.01–2.78
Multiple (> = 3) chronic conditions (=yes)	1.68	1.44–1.97

### Major newborn health profile group

In the ‘major newborn’ health profile group, newborn babies in low-income neighbourhoods were at increased risk of high-cost use – having increased visits to a physician reduced this risk (OR = 1.44 versus OR = 0.81) (Table 7).

**Table 7:** Logistic regression models comparing high-cost health care users and not high-cost use, excluding long-term care residents, by health profile category, Saskatchewan, April 1, 2015 to March 31, 2017 (*n* = 1,175,147). High-cost use within major newborn health profile group, odds ratio (Total *n* = 2678; High cost users *n* = 267)

Focus setting	Comparison setting	OR	95% CI
History of hospitalization (=yes)	History of hospitalization (=no)	1.52	1.13–2.0
Low income = y; High Dr. visits = y	Low income = n; High Dr. visits = y	0.81	0.53–1.25
Low income = y; High Dr. visits = n	Low income = n; High Dr. visits = n	1.44	1.00–2.15

### Other mental health profile group

We aimed to model the costliest health profile groups, in terms of average cost, which would include the ‘major mental health’ group. However, in Saskatchewan, a systematic error in electronic medical databases throughout the province incorrectly defines ICD-9 code 298 as ‘dementia’ as opposed to the correct definition of ‘inorganic psychoses’. Due to this error, the ‘major mental health’ health profile group has a large preponderance of individuals > = 80 years with high health care utilization; likely indicative of dementia patients.

As mental health and addiction is a known driver of high-cost health care use [29], we include results for the ‘other mental health’ health profile group, not one of the costliest groups, but, a representation of a mental health and addictions health profile group.

In the ‘other mental health’ category low income interacted with high physician visits; those with low income and high physician visits had an increased risk of high-cost use (OR = 5.2; 95% CI: 4.8–5.5) compared to individuals not low income with high physician visits (OR = 3.35; 95% CI: 3.02–3.72) (Table 8).

**Table 8:** Logistic regression models comparing high-cost health care users and not high-cost use, excluding long-term care residents, by health profile category, Saskatchewan, April 1, 2015 to March 31, 2017 (*n* = 1,175,147). High-cost users within other mental health profile group (Total *n* = 62,381; High-cost users *n* = 6241)

Focus setting	Comparison setting	OR	95% CI
Home care = y; Hx of hosp = y	Home care = y; Hx of hosp = n	5.72	4.81–5.53
Home care = n; Hx of hosp = y	Home care = n; Hx of hosp = n	11.66	10.52–12.93
Low income = n; High Dr. visits = y	Low income = n; High Dr. visits = n	3.35	3.02–3.72
Low income = y; High Dr. visits = y	Low income = y; High Dr. visits = n	5.16	4.81–5.53

## Discussion

Rising health care costs is a significant challenge for health care systems in Canada and around the world. The current study aimed to understand high-cost health care users from the perspective of a provincial health insurer. Using variables readily available in provincial administrative health databases, in combination with CIHI’s Population Grouping methodology, study results add to the evidence available to decision-makers as they develop policies to reduce costs, and, ultimately, improve the health of this population.

In the majority of health profile groups modelled, a measure of socio-economic status – neighbourhood income quintile – was statistically significantly associated with high-cost use.

Measures of socio-economic status (SES), such as, unstable housing, and food insecurity, have previously been found to be associated with high-cost health care use, [4, 22, 28, 30], To the best of our knowledge this is the first population-based study to define high-cost users within their health profile group and consider SES in regression models. We hypothesize this method provides better specificity to understanding high-cost users of health care services.

Equity in health is of utmost importance. We feel that by providing quantitative evidence demonstrating the association between low socio-economic status and high-cost health care use within specific health profile groups – policy makers can create interventions aimed to both reduce costs and improve health.

A recent study out of the US similarly found that population segmentation methods were useful for defining actionable high-cost user cohorts [11]. The authors defined six cohorts: under 65 years of age and disabled/end-stage renal disease; frail elderly; major complex chronic, minor complex chronic; simple chronic; and relatively healthy. Individuals in the top 10% of spending were high-cost. The authors conclude that using simple criteria that segments, in this case, Medicare beneficiaries, into meaningful subgroups is a useful method to target interventions –a conclusion similar to the current study [11]. Besides only using data specific to one population (Medicare recipients), and, a non-validated approach to segmentation, this study is further limited by the fact that “high-cost use” was defined overall and not within each population segment. In addition, the authors did not conduct analyses beyond descriptive statistics, limiting the ability of their work to point to risk factors associated with high-cost use within the population segments.

Another recent study from the US also described the utility of population segmentation in describing high-cost users. This study employed density based cluster analysis to determine the population segments; however, their analysis did not include any indicators of socio-economic status [16]. A recent systematic review of high-cost health care use, synthesizing 55 studies around the world, indicated that high-cost use was associated with multiple chronic conditions, older age, mental illness, end-of-life care, higher income (United States) and lower income (all other countries). Most relevant to the current work, however, the authors note that given the heterogeneity of high-cost user populations segmentation methods to define specific groups prior to analyses is recommended [29].

Although our study population is limited to one provincial jurisdiction in Canada, the methods used is generalizable and useful for other jurisdictions where a central health insurer is interested in defining their high-cost health care population. High-cost users are not a homogenous group. Segmenting the population into health profile groups shows promise in describing the populations that consume the most health care resources.

Policy and decision-makers require actionable information. Providing descriptive epidemiology on high-cost health care users is not enough – researchers must provide actionable information for policy and decision-makers. The methods and analyses conducted to arrive at a reasonable conclusion about what is driving high-cost health care use are often complex. It is incumbent upon implementation scientists to know how to communicate complex findings in a simple, easy to understand manner, in addition to focusing on what is amenable to change, such as socio-economic status, either at the individual or population-level.

### Limitations

This study has several limitations, many inherent to epidemiological studies reliant on administrative health databases. Additional variables, such as food insecurity [27] have been found to be associated with high-cost use and may have been useful in modelling; however this data was not available for analysis. Community-based services, either publicly funded or private fee-for-service, (individual/group counselling, treatment centres, private nursing services, others) may be associated with high-cost use but not available for analysis. This is one of the largest drawbacks of studies making use of administrative health data – important confounders and predictors are often not available for analysis. Recent model simulation work has identified ways to impute some of these missing variables, though little is known if these methods work in practice [25].

Readers will note that the epidemiological context of this study is somewhat ‘muddy’ – the outcome of ‘high-cost use’ within specific health profile groups can be related to the definition of the group itself (a combination of resource intensity and severity). We acknowledge this less than ideal context, however, we feel the population segmentation method still provides utility.

While we did account for the costs of prescription drugs, we did not examine the types of prescription drugs taken. There may well be an association between drug class and high-cost health care use.

Our study focused on cost; however, we could not measure all all health care system costs, such as cancer treatment/diagnosis, laboratory testing, home care, public health, travel costs (air transfers and ground ambulance) and all health care administrative costs. High-cost users comprised a small proportion (10%) of the study population, yet accounted for 41% of measured health care costs. Given our inability to account for health care costs in previous studies, such as cancer diagnosis/treatment and laboratory costs, our findings are in general agreement with the literature.

In defining comorbidities, we relied on available health condition ‘tags’ in the Pop Grouper software; however, this method has not been validated against a gold standard method of chronic disease indicators (such as the Canadian Chronic Disease Surveillance System) [9]. This study relied heavily on population segmentation, which may not always accurately classify individuals. For example, a person may be a high-cost user due to an opioid addiction, but, be classified into heart disease if the condition comprised their costliest health care utilization. Alternatively, individuals who did have a specific health condition under study, but, did not ever seek medical treatment for it, would be classified as ‘health system non-users’. The segmentation methods may inadvertently not delineate specific groups associated with high-cost use, such as medical error [16].

We were unable to account for the specific days of insurance coverage for individuals who died/moved out of province; however, reassuringly 93% of study cohort members had complete follow-up time. As our study population comprised individuals followed over 24 months we did not consider high-cost health care use that persisted over time – differences in results would likely occur between episodic and persistent high-cost use.

We were limited by underreporting of emergency department visits in Saskatchewan as, during the study period, only < 50% of the provincial emergency departments were NACRS-reporting facilities. Similarly, physician claims data would be limited in missing alternative-payment physician claims that do not shadow bill. Many psychiatrists in Saskatchewan are on alternative-payment schedules; this may disproportionately underestimate the number of individuals in mental health groups.

It should be noted that all of the models, while demonstrating reasonable calibration and validation, had low pseudo R^2^ values. This indicates the variables available for analysis are not explaining the majority of the variation between high-cost use and not high-cost use. The use of additional information not commonly available in administrative health databases, such as, social support, trust in the health care system, individual-level indicators of socio-economic status and others would likely greatly improve model variation explanation.

Costing data was missing for 54,948 individuals in the study population. It is possible that inclusion of those individuals would change model interpretation; however, given the small numbers of missing values (4%) we feel the impact of this limitation is likely to be minimal.

Additionally, high-cost health care use, while concerning from an insurer perspective, is not necessarily an adverse outcome. For example, high-cost health care use in obstetric patients may be entirely appropriate; driven by increased visits to specialists during a high-risk pregnancy and associated with better health outcomes. We were unable to assess quality of life outcomes in this study.

Lastly, ‘high-cost’ use definitions vary between those who exceed the 90th percentile, 95th percentile or 99th percentile of total population costs. We chose to focus on individuals in the top 10% of total costs per fiscal year as the majority of high-cost user studies in the literature (see Introduction) define ‘high-cost use’ as those in the top 10% of costs. However, we acknowledge the arbitrary nature of any cut-off value used.

### Future work

Our finding that ‘palliative’ is one of the costliest health profile groups is not unexpected [2, 19] and warrants further research to understand factors associated with this increased cost.

In only two health profile groups, ‘major acute’ and ‘major cancer’, high-cost use was not associated with lower socio-economic status. The factors associated with high-cost use in these two groups (major acute and major cancer) are health care utilization variables (such as lengthy hospital stays) which would be expected. The number of days in hospital avoided with good continuity of health care providers in the community would be interesting to quantify for major acute and major cancer patients (less expensive use of resources compared to acute in-patient beds).

Future work could also consider high-cost use over time, potentially focusing on groups of trajectories, qualitative inquiries with high-cost users, linking health care costing data with other human services costing data to obtain a complete picture of ‘high-cost use’, and spatial analyses of high-cost use. The two costliest health profile groups in our study were palliative care and long-term care residents; the majority of which considered ‘high-cost users’. A detailed study into end-of-life costs and the reasons behind high-cost use for palliative patients (including specific interventions that not only save costs, but improve quality of life for patients); especially given previous contradictory findings regarding end-of-life costs [2].

## Conclusion

Given the heterogeneity, complexity and the natural ‘regression to the mean’ in health care spending, policy and decision-makers have difficulty devising interventions aimed at reducing high-cost health care use at a population level. By segmenting a provincial population into clinically meaningful sub-populations and demonstrating a link between socio-economic status and high-cost health care use in the majority of health profile groups, but not all, we feel this study adds to the body of evidence aimed at understanding the complexity of high-cost health care use.

Furthermore, this study provides quantitative evidence to support an agenda to improve equity in health. Within the majority of health profile groups, and taking into account a myriad of potentially confounding variables, individuals with lower socio-economic status were more likely to be high-cost health care users. Perhaps, both improved population health and cost savings could be achieved if policy makers addressed the underlying inequity.

By grouping the study population into health profiles, and understanding factors associated with high cost use within each specific health profile, the evidence generated becomes more specific, and, potentially actionable. It is impossible to intervene at a system level on an individual’s age, but system level interventions can be actioned and directed at improving socio-economic status, managing multiple comorbid conditions and improving end-of-life care.

There are relatively few examples of interventions that have successfully both reduced costs and improved health outcomes with respect to high-cost users. InterMountain Health Care in Utah and the Camden Primary Care consortium in New Jersey have achieved improved health outcomes and cost savings by focussing on the few consuming the most resources. In both instances, high-cost individuals were a homogeneous group; in New Jersey the high-cost, high-utilization population was concentrated in a small geographical area and were of lower socio-economic status [6]. In Utah, the Connected Care Clinic was designed to serve the ‘complex few’ – those with multiple chronic conditions and multiple psycho-social issues (such as unstable housing and food insecurity). By ‘wrapping services around’ these complex few InterMountain was able to achieve positive results [18, 30].

Understanding the demographics, clusters, health care utilization patterns and predictors associated with high-cost health care use will be important for identifying opportunities for upstream prevention. Providing more targeted, appropriate care and supports for specific sub-populations, such as, mental health, newborns or multi-morbid individuals, or, by acting on the determinants of health to prevent certain types of high-cost use in the first place could achieve positive results. For example, we found that for babies born in low-income neighbourhoods, increased physician visits decreased the risk of high-cost use. Related policies could include identifying at risk pregnant mothers and connecting them to primary care providers prior to the birth of their baby. Similarly, home care services could be a priority for mental health patients coming out of hospital. Given high-cost use for most groups was associated with low socio-economic status, we could provide disadvantaged/vulnerable persons with major chronic disease with one-on-one social worker support – decreasing health care costs and improving quality of life. We feel that by developing well calibrated and discriminatory models aimed at understanding factors associated with high-cost use we are providing a piece of the puzzle for policy makers keen to implement interventions. If successful, these interventions could both lower costs, but more importantly, improve the health of the population.

## Supplementary information

**Additional file 1:** CIHI Population Grouping Methodology.

## Data Availability

Aggregate data available upon request.

## Abbreviations

Dr.: Doctor
Dx: Disease
Hosp: Hospitalization
Hx: History
LOS: Length of hospital stay
Multi chronic: Multiple (> = 3) chronic conditions
N: No
Y: Yes
