# Supplementary material for: Defining ‘actionable’ high- costhealth care use: results using the Canadian Institute for Health Information population grouping methodology
Source: Int J Equity Health. 2019 Nov 10;18:171. doi: 10.1186/s12939-019-1074-3 (PMC6842471; doi:10.1186/s12939-019-1074-3)
Supplement: Supplementary file 1 — Additional file 1: CIHI Population Grouping Methodology. [file 12939_2019_1074_MOESM1_ESM.docx]

## Additional file

### S1: CIHI Population Grouping Methodology

CIHI’s population grouping methodology uses a combination of administrative health databases and provincial health registry systems to ‘tag’ each resident with any of the 239 health conditions. These binary tags (0/1) form the ‘building block’ of the grouping methodology and are not mutually exclusive; an individual can have any number of applicable health conditions. The presence of health conditions are determined by linking data from hospitalizations, physician visits, hospital day surgeries, emergency department visits and long-term care. The method uses 24 months of data to determine health conditions (n = 239), branches (n = 164) and health profile groups (n = 16).

Groups of health conditions that are clinically related are addressed using ‘clinical overrides’. Each of the clinical scientific working groups developed clinical overrides. Overrides were designed to reduce redundancies and are composed of the following rules: 1) More severe manifestations of disease override less severe, 2) Overrides ‘fix’ differences in provincial coding practices (for example, ‘influenza’ will override ‘unspecified infection’ , and, 3) Diagnosis will override symptoms (for example, acute gastrointestinal hemorrhage will override abdominal pain when concurrent).

Physician billing data has specific health condition tagging rules. Physician billing data in Canada has known limitations, largely due to the ability to code for only one three-digit diagnosis, and, the fact that this data source is not subject to rigorous, national data quality initiatives as other administrative health databases. For some conditions identified through physician data only, the condition needs to occur more than once over the 24-month period in order to ‘count’ as a health condition. For example, for the ICD-9 code 300, ‘anxiety’, if coded in the physician billing data would only ‘tag’ an individual with the anxiety health condition if it occurred >= 2 times within the 24 month period in the physician data, and, never in any other data sources. The purpose of these physician data only rules was to minimize false positive health conditions. All physician data rules fall into one of the following categories, based on specific diagnosis: 1) one diagnosis needed to ‘count’, 2) >= 2 physician visits on two or more separate dates, or, 3) no diagnosis needed (for example, ‘healthy newborn’ assigned based on age and/or hospitalization, no physician visit is required to ‘count’).

Next, each of the 239 health conditions are grouped into 164 branches and ranked in order from most severe to least severe. Each technical working group, including clincians, created the severity rankings. Severity was assessed based on two criteria, clinical judgement (clinical complexity) and average health care costing data (resource intensity).

The branches compile related health conditions into the same ‘branch’; branches often split based on the presence or absence of significant comorbid conditions. ‘Significant’ comorbid conditions were determined through descriptive data analyses with input from clinical expert working groups; assessment consisted of two categories: 1) If sufficient numbers of individuals with or without comorbid conditions existed, and, 2) If there was a substantial difference in cost between individuals with or without comorbid conditions. Similar to health conditions, branches are not mutually exclusive.

Each of these branches link to one of 16 mutually exclusive health profile groups. Where an individual belongs to more than one branch, the one with the largest severity/resource intensity determines health profile group. Health profile group severity, as with health conditions, was determined based on a combination of clinical judgement and resource intensity. Each of the mutually exclusive health profile groups are ranked in order of severity (palliative care group as the most severe; health care non-users as least severe).

Figure S1: Health profile groups (n = 16) in order of severity, CIHI Population Grouping methodology

### S2: Effect estimates

Odds ratios values are reported in ‘Results; all effect estimates by health profile group follow.

Table S1: High-cost use within major chronic health profile group, effect estimates (Total n = 37,803; High-cost users n = 3,781)

| **Effect** | **β** | ***SE* β** | ***p-*value** |
| --- | --- | --- | --- |
| Home care  (=yes) | 0.67 | 0.04 | <0.0001 |
| Length of hospital stay >= 3 days  (=yes) | 2.73 | 0.09 | <0.0001 |
| Low income  (=yes) | -0.50 | 0.23 | 0.134 |
| Length of hospital stay >= 3 days X Low income  (yes, yes) | 0.66 | 0.23 | 0.004 |
| High physician visits  (=yes) | 0.82 | 0.19 | <0.0001 |
| Multiple (>=3) chronic conditions  (=yes) | -0.20 | 0.26 | 0.144 |
| High physician visits X Multiple (>=3) chronic conditions  (yes, yes) | 0.80 | 0.27 | 0.003 |
| *Intercept* | -5.90 | 0.19 |  |

Pseudo R^2^= 0.27; Hosmer and Lemeshow Goodness-of-Fit: χ^2^=14.4; df=8; *p*=0.1

Table S2: High-cost users within moderate chronic health profile group, effect estimates (Total n = 112,776; High-cost users n = 11,277)

| **Effect** | **β** | ***SE* β** | ***p-*value** |
| --- | --- | --- | --- |
| Home care  (=yes) | 0.71 | 0.03 | <0.001 |
| Mental health condition  (=yes) | 0.30 | 0.03 | <0.001 |
| History of hospitalization  (=yes) | 1.40 | 0.02 | <0.001 |
| Low income  (=yes) | -0.10 | 0.04 | 0.510 |
| History of hospitalization X Low income  (yes, yes) | 0.16 | 0.05 | 0.003 |
| *Intercept* | -2.78 | 0.02 |  |

Pseudo R^2^= 0.10; Hosmer and Lemeshow Goodness-of-Fit: χ^2^=8.3; df=4; *p*=0.1

Table S3: High-cost use within major newborn health profile group, effect estimates (Total n = 2,678; High cost users n = 267)

| **Effect** | **β** | ***SE* β** | ***p-*value** |
| --- | --- | --- | --- |
| History of hospitalization  (=yes) | 0.41 | 0.15 | 0.005 |
| Low income  (=yes) | 0.37 | 0.20 | 0.602 |
| High physician visits  (=yes) | 1.23 | 0.17 | <0.001 |
| Low income X High physician visits  (yes, yes) | -0.58 | 0.3 | 0.054 |
| *Intercept* | -2.91 | 0.12 |  |

Pseudo R^2^=0.10; Hosmer and Lemeshow Goodness-of-Fit: χ^2^=3.23; df=5; *p*=0.67

| **Effect** | **β** | ***SE* β** | ***p-*value** |
| --- | --- | --- | --- |
| Home care  (=yes) | 0.90 | 0.07 | <0.0001 |
| Low income  (=yes) | 0.52 | 0.05 | <0.0001 |
| History of hospitalization  (=yes) | 2.46 | 0.05 | <0.0001 |
| High physician visits  (=yes) | 1.64 | 0.04 | <0.0001 |
| Home care X History of hospitalization  (yes, yes) | -0.71 | 0.18 | <0.0001 |
| Low income X High physician visits  (yes, yes) | -0.43 | 0.06 | <0.0001 |
| *Intercept* | -3.25 | 0.03 |  |

Table S4: High-cost users within other mental health profile group, effect estimates (Total n = 62,381; High-cost users n = 6,241)

Pseudo R^2^= 0.15; Hosmer and Lemeshow Goodness-of-Fit: χ^2^=9.4; df=4; *p*=0.1

| **Effect** | **β** | ***SE* β** | ***p-*value** |
| --- | --- | --- | --- |
| Length of hospital stay >= 3 days  (=yes) | 3.57 | 0.14 | <0.0001 |
| High physician visits  (=yes) | 1.84 | 0.17 | <0.0001 |
| Multiple (>=3) chronic conditions  (=yes) | 1.32 | 0.26 | <0.0001 |
| Multiple (>=3) chronic conditions X High physician visits  (yes, yes) | -0.59 | 0.26 | 0.025 |
| *Intercept* | -7.39 | 0.21 |  |

Table S5: High-cost users within major acute disease health profile group, effect estimates (Total n=34,733; High cost users n=3,473)

Pseudo R^2^=0.26; Hosmer and Lemeshow Goodness-of-Fit: χ^2^=0.6; df=4; *p*=0.97

Table S6 High-cost users within major cancer health profile group (Total n=8,711; High-cost users n=872)

| **Predictor** | **β** | ***SE* β** | ***p-*value** |
| --- | --- | --- | --- |
| Length of hospital stay >= 3 days (=yes) | 3.14 | 0.26 | <0.0001 |
| High emergency department visits (=yes) | 0.46 | 0.08 | <0.0001 |
| History of hospitalization (=yes) | 0.48 | 0.13 | 0.0001 |
| Home care (=yes) | 0.86 | 0.08 | <0.0001 |
| Multiple (>=3) chronic conditions (=yes) | 0.52 | 0.08 | <0.0001 |
| *Intercept* | -6.16 | 0.26 |  |

Pseudo R^2^=0.25; Hosmer and Lemeshow Goodness-of-Fit: χ^2^=7.01; df=8; *p*=0.54
